# Protoplast‐Based Functional Genomics and Genome Editing: Progress, Challenges and Applications

**DOI:** 10.1111/pce.70375

**Published:** 2026-01-11

**Authors:** Jo‐Wei Allison Hsieh, Fu‐Hui Wu, Dian‐Xuan Yang, Ai‐En Wu, Ching‐Ann Liu, Chang‐Hung Chen, Shinn‐Zong Lin, Ying‐Chung Jimmy Lin, Choun‐Sea Lin

**Affiliations:** ^1^ The Genome Center University of California, Davis Davis California USA; ^2^ Agricultural Biotechnology Research Center Academia Sinica Taipei Taiwan; ^3^ Institute of Plant Biology, College of Life Science National Taiwan University Taipei Taiwan; ^4^ Neuroscience Center, Buddhist Tzu Chi Hospital Hualien Taiwan; ^5^ Department of Medical Research Buddhist Tzu Chi Hospital Hualien Taiwan; ^6^ Department of Neurosurgery Hualien Tzu Chi Hospital Hualien Taiwan; ^7^ Department of Life Science, College of Life Science National Taiwan University Taipei Taiwan; ^8^ Genome and Systems Biology Degree Program National Taiwan University and Academia Sinica Taipei Taiwan

**Keywords:** CRISPR, regeneration, single‐cell transcriptome, transgene‐free gene editing

## Abstract

Protoplast‐based systems provide a powerful and versatile platform for exploring how plants sense and respond to their environment. By enabling the direct delivery of proteins, DNA, and RNA into plant cells after cell wall removal, this approach facilitates precise molecular dissection of signaling, stress adaptation, and gene regulation across both model species and economically important crops. In this review, we analyzed 1050 published articles and categorizing them by delivery methods, research focus, plant species, and tissue types. We further highlight recent advances, including the application of single‐cell transcriptomics, which provides unprecedented resolution for dissecting cellular responses and offers deeper insights into the mechanisms underlying stress resilience. Importantly, protoplast regeneration is gaining renewed attention not only as a model system for studying cellular reprogramming but also as a practical platform for crop improvement. Applications of protoplast regeneration include protoplast fusion, which integrates nuclear and organellar DNA/genomes from divergent parents to accelerate breeding and enhance tolerance to both biotic and abiotic stresses. Another important application is CRISPR/Cas ribonucleoprotein (RNP)‐based editing targeting stress‐resilience‐related genes. In asexually propagated or highly heterozygous perennial crops with limited sexual reproduction, protoplast‐based RNP delivery offers a viable and regulation‐compliant strategy. This approach may help address public concerns over transgenic technologies while enabling the rapid development of stress‐tolerant cultivars.

## Summary statement

- Based on an analysis of 1050 studies, this review shows that protoplast‐based systems have evolved into a powerful platform for both mechanistic studies and crop improvement, integrating advances such as single‐cell transcriptomics, protoplast fusion, and CRISPR/Cas RNP editing to enable precise and regulation‐compliant development of stress‐resilient crops.

## 1 Introduction

**Figure 1:**
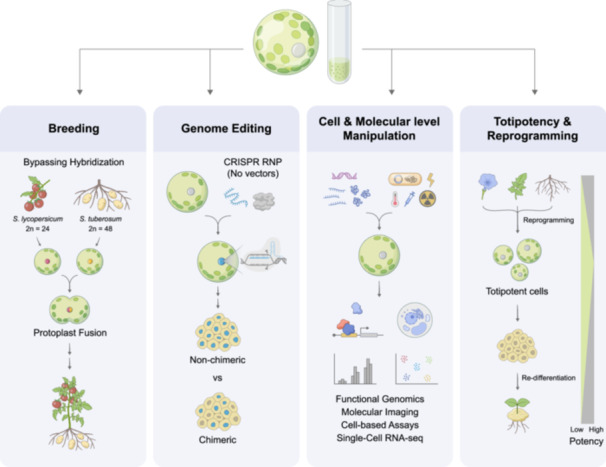
Applications of protoplasts in plant biotechnology. Protoplasts serve as a versatile platform for various applications in modern plant science. Breeding: In plant breeding, protoplast fusion allows interspecific hybridization between sexually incompatible species. For example, fusion between *Solanum lycopersicum* and *S. tuberosum* allows the introduction of traits that cannot be achieved through conventional crossing, and the same principle has also been applied in other *Solanum* species, such as *S. peruvianum* (Chen and Adachi 1998). Genome Editing: In genome editing, CRISPR ribonucleoprotein (RNP) complexes can be delivered without vectors, enabling precise, non‐chimeric edits at the single‐cell level. Cell & Molecular level Manipulation: Protoplasts enable manipulation at the cellular and molecular levels, including applications in functional genomics (e.g., promoter activity, protein subcellular localization and protein–protein interaction tests), molecular imaging (e.g., live‐cell imaging of hormone responses, calcium waves), cell‐based assays, and single‐cell RNA sequencing. Totipotency & Reprogramming: Protoplasts exhibit high totipotency. After cell wall removal, differentiated cells can dedifferentiate into callus and then re‐differentiate into shoots and roots, eventually regenerating into whole plants under appropriate culture and hormone regimes. This regeneration process provides an experimental framework to study cell fate reprogramming, chromatin remodeling, epigenetic resetting, and developmental plasticity across genotypes and tissue sources.

Rapid climate change presents a pressing challenge: to elucidate the mechanisms underlying crop stress tolerance and translate this knowledge into the development of climate‐resilient cultivars that comply with regulatory frameworks, align with societal acceptance, and minimize environmental harm while securing global food supplies. Since the establishment of plant protoplast technology, it has become a key tool for overcoming the limitations of traditional breeding. In particular, the development of somatic cell fusion has enabled the fusion of protoplasts from different species, followed by plant regeneration through tissue culture, thereby bypassing sexual incompatibility barriers. This technique not only facilitates the creation of somatic hybrids but also offers a new approach for introducing beneficial traits, such as those conferring tolerance to drought, salinity, disease resistance, and other environmental stresses, from wild species into cultivated crops without relying on conventional sexual reproduction (Figure 1, Breeding). With advances in regeneration and gene manipulation techniques, protoplast technology has become an increasingly valuable tool in modern plant biotechnology, demonstrating its unique advantages (Yue et al. 2021).

One major application is genome editing, particularly in modifying stress‐related genes. Removal of the cell wall facilitates the direct delivery of Clustered Regularly Interspaced Short Palindromic Repeats/CRISPR‐associated proteins (CRISPR/Cas) reagents into the protoplasts, which improve editing efficiency and circumvent the need for vector‐based transformation (Yue et al. 2021). Conventional gene editing approaches frequently rely on transgene integration to deliver CRISPR/Cas reagents (Graham et al. 2020; Gao 2021), which not only suffers from low efficiency and limited applicability across plant species but also raises regulatory, environmental, and public concerns associated with the use of genetically modified organisms (GMOs), potentially constraining downstream applications. Additionally, the single‐cell nature of protoplasts provides a controlled system for introducing precise genetic modifications and regenerating uniform, edited plants from individual cells, thereby avoiding mosaicism and ensuring the development of genetically stable lines (Yue et al. 2021; Scintilla et al. 2022; Figure 1, Genome Editing).

Another key strength lies in the ability to access plant cells in a cell wall‐free state, allowing for precise manipulation at both the molecular and cellular levels, they can be used to rapidly test gene expression constructs, promoters, regulatory elements, and synthetic circuits before applying them to whole plants. This enhanced accessibility facilitates not only the delivery of proteins, DNA, RNA, and ribonucleoproteins, but also the uptake of dyes, probes, and nanoparticles—making protoplasts a highly versatile system for functional genomics, molecular imaging, and various cell‐based assays (Figure 1, Cell & Molecular Level Manipulation). Protoplasts also serve as an ideal system for high‐throughput screening (Lee et al. 2012; Yeh et al. 2019). This significantly reduces the time and resources needed for construct validation. Moreover, protoplasts offer a highly controlled and homogeneous cellular system that enables the dissection of complex hormonal crosstalk, signal transduction cascades, transcriptional regulation, and stress‐responsive networks under precisely defined environmental conditions. Such precision facilitates the identification of subtle, early‐stage molecular events and key regulatory genes underlying plant responses to environmental challenges, providing unique opportunities to unravel the intricate mechanisms linking cellular signaling with whole‐plant adaptation.

From a developmental biology perspective, protoplast culture and regeneration also offer a valuable platform for investigating cell totipotency and reprogramming. Observing how a single differentiated cell reverts to a pluripotent state and regenerates into a whole plant provides insights into fundamental mechanisms of cellular plasticity, particularly how plants, when challenged by environmental stresses, can reconfigure their growth and developmental programs to enhance survival and adaptation (Figure 1, Totipotency & Reprogramming). Altogether, protoplasts represent a flexible, high‐resolution, and experimentally tractable system that bridges molecular manipulation and whole‐plant outcomes—supporting innovation across both basic and applied plant science (Figure 1).

## 2 Meta‐Analysis of 1050 Studies on Protoplast Transient Systems: Global Patterns and Applications

**Figure 2:**
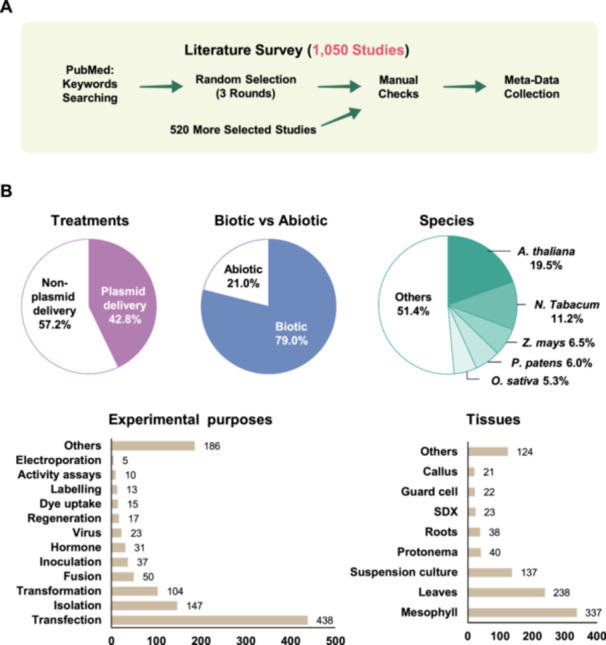
Meta‐analysis of 1050 published studies on plant protoplast applications. (A) Overview of the literature survey pipeline. A keyword‐based search on PubMed retrieved more than 13 000 publications. The data set was refined through three rounds of random selection, followed by the addition of 520 targeted studies identified from focused curation. After manual screening, 1050 publications were retained. Metadata from these studies were then systematically extracted and organized for downstream comparative analyses. (B) Summary of key characteristics across studies. The upper panel presents three pie charts summarizing the distribution of treatments by classifying them into plasmid‐delivery and non‐plasmid‐delivery approaches (Upper left), the proportion of biotic versus abiotic studies (Upper middle), and the top species represented in the survey (Upper right). The lower panel contains two bar charts that illustrate the major experimental purposes (Lower left) and the tissue types examined (Lower right). Exact counts are displayed above each bar to indicate the underlying sample sizes. [Color figure can be viewed at wileyonlinelibrary.com]

To comprehensively evaluate the global application and methodological diversity of protoplast‐based transient expression systems, we systematically curated and analyzed 1050 peer‐reviewed publications (Figure 2A and Table S1). The papers were randomly selected from keyword searches on PubMed across three iterative rounds, each initiated after completing manual screening of the previous batch, until sufficient coverage of protoplast‐related studies was achieved. Our analysis focused on four key dimensions: (1) the diversity of treatments and experimental techniques, (2) the range of plant species utilized, (3) the types of biological or environmental stimuli applied, and (4) the tissue sources used for protoplast isolation. Together, these dimensions provide a quantitative overview of how protoplast platforms are being employed across plant research and breeding disciplines, highlighting both current trends and gaps in the literature.

We also analyzed the types of treatments and tools used in protoplast‐based transient expression studies. Among the 1000 collected publications, plasmid delivery remains the most widely adopted approach, appearing in nearly half of all studies (481 counts; Figure 2B). This reflects its long‐standing use for transient gene expression analysis, subcellular localization, and promoter activity assays. Following this, CRISPR‐based genome editing—particularly through DNA‐free delivery of ribonucleoprotein (RNP) complexes—has rapidly gained traction in recent years, ranking as the second most frequent treatment recorded. Other common entries include the use of fluorescent proteins, polyethylene glycol (PEG)/Ca^2+^‐mediated transfection, and various marker genes or reporter constructs. Interestingly, a large diversity of experimental inputs was observed, ranging from cell‐penetrating peptides and nanoparticles to plant hormones, transcription factors, and stress‐related signaling molecules (Supporting Information S1: Figure S1). This diversity highlights the versatility of the protoplast transient system as a platform not only for genome editing, but also for dissecting signaling cascades, evaluating protein function, and conducting synthetic biology assays at the single‐cell level.

To further evaluate the functional diversity of protoplast‐based applications, we categorized the experimental purposes across the 1000 studies. The most frequently observed applications were transfection (438 studies), isolation (147), transformation (104), and fusion (50) (Figure 2B), indicating that protoplast systems are widely used not only as recipients for gene delivery, but also as core components of broader genetic engineering workflows. These were followed by studies involving inoculation (37), hormone response assays (31), and virus‐based delivery (23), underscoring the versatility of protoplasts in both biotic and abiotic interaction studies. Interestingly, the dataset revealed a long tail of experimental diversity, including regeneration (17), CRISPR‐mediated editing, dye uptake (15), labelling (13), activity assays (10), and electroporation (5) (Figure 2B). A wide array of environment‐related treatments—such as temperature, gravity, toxins, antibiotics, and light—were also explored, often in combination with fluorescent markers or transient gene expression reporters (Supporting Information S1: Figure S2). These findings highlight that, beyond genome editing, protoplast systems are increasingly used to interrogate cellular responses, signaling mechanisms, and environmental interactions at high resolution.

To examine species usage trends, we compiled the genus and species information from all studies. A total of 234 distinct species were identified, reflecting the broad taxonomic application of protoplast‐based transient systems. However, the distribution was highly skewed toward a small number of model species. *Arabidopsis thaliana* was the most frequently used species (239 studies), followed by *Nicotiana tabacum* (137), *Zea mays* (80), *Physcomitrella patens* (73), and *Oryza sativa* (65) (Figure 2B). These five species accounted for more than 48.6% of the studies, highlighting their central role in the development and optimization of protoplast systems. Aside from model plants, several important crop species such as *Glycine max*, *Triticum aestivum*, *Z. mays*, *Solanum lycopersicum*, and *Brassica napus* also appeared frequently, reflecting efforts to extend protoplast techniques into economically important species (Supporting Information S1: Figure S3). Interestingly, many other species—including trees, non‐vascular plants, and ornamentals—were represented by only one or two publications (Supporting Information S1: Figure S3). This long‐tail distribution underscores both the adaptability of protoplast technology across taxa and the need for continued optimization in less‐studied species.

We further analyzed the tissue sources used for protoplast isolation across the selected studies. Among the diverse range of tissues reported, mesophyll cells were by far the most commonly used source (337 studies), followed by general leaf tissues (238) and suspension cultures (137). These results align with the historical reliance on leaf‐derived protoplasts in model systems such as *A. thaliana* and *N. benthamiana*, where protoplast yields are high and protocols are well established. Other tissues, such as roots (38), stem‐differentiating xylem (23), guard cells (22), and callus (21), were used far less frequently but suggest a growing interest in exploring cell type‐specific responses and differentiation states (Figure 2B). Notably, a long tail of tissue types—including pollen, aleurone, embryos, and cambium (Supporting Information S1: Figure S4)—were represented by only a few studies each, highlighting both the versatility and the current technical limitations in isolating protoplasts from highly specialized or deeply embedded tissues.

To better understand the functional applications of protoplast‐based transient systems, we further categorized the treatments into biotic and abiotic types (Figure 2B). Among the studies analyzed, biotic treatments were overwhelmingly dominant, accounting for 665 out of 842 classified entries (~79%) (Supporting Information S1: Figure S5). These treatments include pathogen infection, virus inoculation, microbial elicitors, and biotic stress responses (Supporting Information S1: Figure S2 and Table S1), highlighting the widespread use of protoplasts in studying plant–microbe interactions and immune signaling pathways. In contrast, abiotic treatments were reported in only 177 studies (~21%) (Supporting Information S1: Figure S5), including responses to salt, drought, cold, heat, hormone treatments, and various chemical signals (Supporting Information S1: Figure S2 and Table S1). Although less frequently explored, these studies illustrate the growing interest in using protoplasts to dissect abiotic stress responses at the single‐cell level, where environmental signaling pathways can be examined with high temporal and spatial resolution.

Our meta‐analysis reveals that protoplast‐based transient systems are predominantly applied in a handful of model species and are most widely utilized in studies involving biotic stress, gene delivery, and cell‐based functional assays. The plasmid‐based transfection remains the most common delivery method. Despite the concentration of research in specific taxa and techniques, the presence of over 234 plant species, ranging from major crops to non‐model taxa, underscores the broad potential of the platform. Moreover, tissue source analysis shows a strong reliance on mesophyll and leaf tissues, which together account for the majority of protoplast isolations. However, the inclusion of specialized tissues such as roots, callus, suspension cultures, guard cells, and even stem‐differentiating xylem indicates growing interest in expanding the biological contexts in which protoplast systems are applied. Continued methodological innovation—particularly in regeneration protocols, tissue‐specific isolation, and DNA‐free genome editing—will be essential to further expand the utility of protoplasts across diverse plant systems and developmental stages.

## 3 Protoplasts as an Enabling Platform for Plant Single‐Cell Transcriptomics

One of the most significant recent advances in plant molecular biology is the adaptation of single‐cell RNA sequencing (scRNA‐seq), which allows researchers to resolve gene expression at cellular resolution (Tung et al. 2023; Chen, Hsieh, et al. 2024; Hsieh et al. 2025). Unlike animal systems, where dissociating cells from tissues is relatively straightforward, the rigid and complex structure of plant cell walls presents a major obstacle for isolating viable single cells. Protoplasts circumvent this challenge by enzymatically removing the cell wall, allowing for the generation of single‐cell suspensions compatible with high‐throughput scRNA‐seq platforms.

The adoption of protoplast‐based scRNA‐seq has enabled researchers to uncover the transcriptional landscapes of diverse plant tissues at unprecedented resolution. By capturing gene expression profiles from thousands of individual cells, this approach has revealed cell fate transitions, tissue differentiation processes, and dynamic gene regulatory networks underlying stress responses, hormone signaling, and morphogenesis—insights that were previously masked in bulk RNA‐seq datasets. In particular, protoplast‐derived single‐cell data have clarified the spatial organization of gene expression and cell‐type–specific expression of key regulatory genes.

While traditional studies on plant development have relied heavily on anatomical sections and reporter lines, the emergence of protoplast‐mediated scRNA‐seq has provided a powerful new approach to study dynamic developmental processes at high resolution and even to capture reprogramming under environmental stress (Tung et al. 2023; Chen, Hsieh, et al. 2024; Hsieh et al. 2025). Plant development can be broadly conceptualized along four spatial axes—apical‐basal (up‐down) and radial (inside‐outside). For the apical‐basal axis, extensive progress has been made through live imaging of shoot and root apical meristems, which particularly are transparent in model species such as *A. thaliana*, where fluorescent protein reporters allow for in vivo tracking of stem cell dynamics and tissue patterning over time. In contrast, investigating the radial axis of development—especially vascular tissue formation—has been far more challenging. The vascular cambium, which gives rise to both xylem and phloem, is embedded deep within the stem and hidden beneath outer tissue layers, making it inaccessible to standard live imaging techniques. Historically, researchers have had to rely on static cross‐sections—transverse, radial, and tangential—taken at various developmental stages to infer the dynamics of cambium activity and xylem differentiation (Tung et al. 2023; Chen, Hsieh, et al. 2024; Hsieh et al. 2025). This indirect approach has led to the proposal of multiple, often conflicting, models of vascular development.

The application of protoplast‐based scRNA‐seq overcomes this limitation by capturing dynamic transcriptomic changes from individual cells within vascular tissues. Rather than relying on static anatomy, researchers can now reconstruct developmental trajectories based on transcriptional states, revealing the continuous and directional progression of xylem differentiation (Tung et al. 2023; Chen, Hsieh, et al. 2024; Hsieh et al. 2025; Shuai et al. 2025). This approach has already led to the establishment of a widely accepted working model for xylem development under normal conditions and mechanical stress, derived from high‐resolution single‐cell data (Tung et al. 2023; Chen, Hsieh, et al. 2024; Hsieh et al. 2025). Thus, protoplast‐mediated scRNA‐seq is not only a technical breakthrough but also a conceptual advance—providing a much‐needed window into the hidden dynamics of radial development in plants.

To illustrate the current landscape of protoplast‐enabled scRNA‐seq in plants, we curated a set of representative studies that explicitly employed protoplast isolation for single‐cell analysis. Rather than aiming for an exhaustive list, we selected key publications that reflect the diversity of organs, species, and experimental designs. The studies span a range of plant tissues—including anther, flower, maize ear, and shoot—and cover both model and non‐model species such as *Gossypium hirsutum*, *N. attenuata*, *Z. mays*, and *Pisum sativum* (Table S2). These studies reported a wide array of cluster annotations, including vasculature‐ and xylem‐related cell types, with several also reconstructing developmental trajectories from the single‐cell data. This curated collection demonstrates how protoplast‐based methods are advancing plant developmental biology and cell‐type classification at high resolution.

The flexibility of the protoplast system further enhances its value, allowing researchers to isolate cells from specific tissues such as mesophyll, root, shoot, meristem, or reproductive organs depending on the biological question. This versatility is consistent with the patterns we observed in our meta‐analysis of 1050 studies (Figure 2B), where mesophyll and leaf tissues were most common, but a growing number of studies successfully employed more specialized tissues such as guard cells, stem‐differentiating xylem, and callus. As single‐cell technologies continue to evolve—particularly with the advent of spatial transcriptomics and multi‐omic integration—protoplasts are poised to remain a foundational component for linking gene function to phenotype at cellular resolution. Their role in single‐cell biology not only complements genome editing and regeneration efforts but also provides a vital bridge from molecular discovery to practical application in plant biotechnology.

## 4 Protoplasts Meet Breeding: From Cell to Field

**Figure 3:**
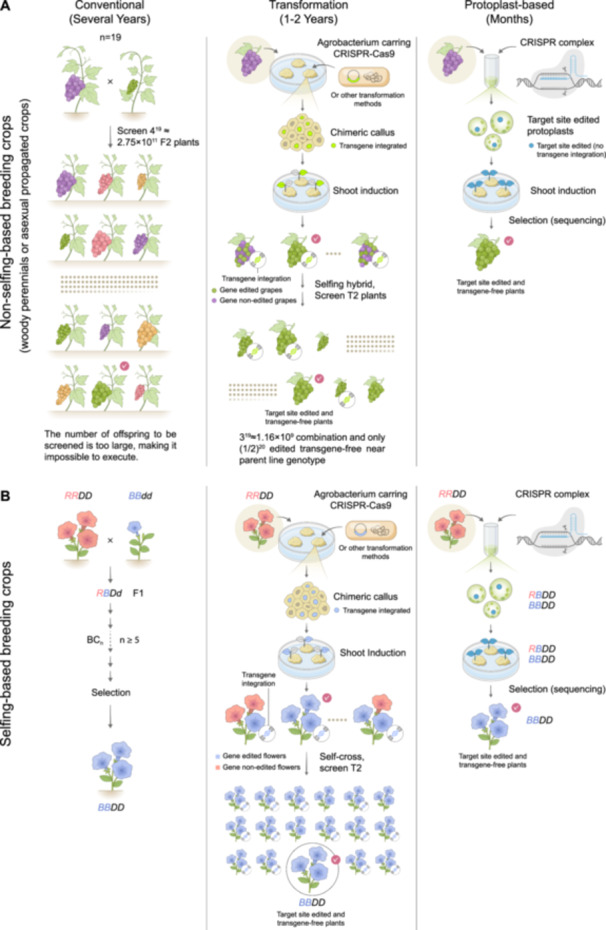
Comparison of conventional, transformation‐based, and protoplast‐based breeding strategies in different crop systems. (A) Asexually propagated crops (e.g., grapevine). (Left) Conventional breeding involves multiple years of crossing and selection to recover desired traits from highly heterozygous offspring. The vast number of possible genetic combinations makes the process inefficient. For example, obtaining a large green grape by crossing a large purple variety with a small green variety. If both parents are heterozygous at 19 relevant loci (*n* = 19), the number of possible F₂ genotypes becomes 4^19^, which is extraordinarily large. Screening such a population is impractical in terms of field space, labor, and time, meaning that recovering the desired combination could require many years of repeated planting and selection. (Middle) Transformation‐based approaches shorten the timeline but often result in chimeric plants or transgenic insertions, requiring further generations to remove unwanted sequences and restore the original genotype. For example, when a ‘green‐skin’ gene is delivered into a large purple‐berry cultivar, the resulting callus commonly becomes chimeric, with only some cells integrating the transgene. After shoot induction, regenerated plants may display the desired large green‐berry phenotype yet still retain Cas9 or vector fragments. Self‐pollination and segregation in the F₂ generation are required to obtain uniform individuals that both carry the target allele and are free of unwanted transgene insertions. Thus, even though transformation speeds up the initial trait introduction, additional generational steps are needed to recover a clean, stable genotype. (Right) Protoplast‐based genome editing enables direct and precise edits in single cells, generating uniform, edited, and transgene‐free plants in a single step within months. (B) Inbred, sexually propagated crops. (Left) Conventional breeding relies on multiple rounds of hybridization, backcrossing, and selection to stack desired alleles while restoring the elite genetic background. For example, crossing an *BBdd* donor with a *RRDD* elite line generates an F₁ genotype of *RBDd*. Introducing the *R* and *D* alleles into the elite *BB* background typically requires at least five successive backcrosses (BC₁–BC₅), selecting each generation for plants that maintain *R* and *D* while restoring the recurrent *BB* genome. After multiple cycles, a near‐isogenic line with the target *BBDD* configuration is finally obtained. (Middle) Transformation introduces target traits but may involve integration of transgenes, which must be segregated out in subsequent generations. In this example, the *BB* allele is introduced into an *RRDD* background using *Agrobacterium* carrying a CRISPR–Cas9 construct. The transformation produces a chimeric callus, with only a subset of cells integrating the editing cassette. Following shoot induction, regenerated plants may carry the edited *B* allele while still retaining Cas9 transgene fragments. Self‐pollination is required to segregate away the transgene, allowing the recovery of clean *BBDD* individuals in the next generation. (Right) Protoplast‐based editing allows direct modification of the elite line, enabling rapid generation of homozygous, edited plants without crossing or prolonged selection. [Color figure can be viewed at wileyonlinelibrary.com]

One of the major goals in plant breeding is to combine desirable traits that exist separately in different individuals into a single, superior genotype. The most traditional and straightforward approach to combining desirable traits is hybridization, which introduces traits from different individuals into a single genotype through sexual crossing (Figure 3A; left panel). However, traditional breeding usually encounters three major challenges: (1) Desirable traits are often not stably inherited through sexual reproduction, especially in crops with high genomic heterogeneity (Mahmoud et al. 2022; Su et al. 2023; Hsu et al. 2024; Figure 3A); (2) Some species are incapable of sexual reproduction, such as *Musa* spp. and *S. tuberosum*, due to factors like polyploidy or sterility (Andersson et al. 2017; Cheng et al. 2024); (3) Desired traits are not only found across different lines within the same species, but may also originate from entirely different species. Hybridization becomes unfeasible when the genetic distance between species is too great, making it impossible to cross certain taxa through conventional means (Li et al.^2020^; Mino et al. 2022; He et al. 2023)

To overcome the limitations of traditional breeding methods, protoplasts have been utilized from various perspectives:

### 4.1 Protoplast Technologies: Overcoming Traditional Barriers

#### 4.1.1 Protoplast Fusion for Enhancing Plant Stress Resistance

Plants are continually challenged by both biotic and abiotic stresses. Developing cultivars that can withstand these conditions is crucial for global food security under rapid climate change. Protoplast fusion, which bypasses the barriers of conventional hybridization, together with wide hybridization strategies, offers complementary tools to accelerate breeding for stress resilience. Since 1980s, the advancement of breeding techniques and the emergence of somatic fusion technology, plant protoplasts had been extensively manipulated through somatic cell fusion followed by tissue culture‐based regeneration techniques to circumvent the hybridization barriers. Such platform facilitates the development of superior traits and the creation of novel polyploid species through fusion. In addition to bypassing hybridization barrier, plant biologists envision the emergence of F1 vigor in plants regenerated from fused cells (Figure 1, Breeding; Power et al. 1970; Melchers et al. 1978; Yue et al. 2021).

For example, in citrus, the fusion line ‘G1 + HBP’ was developed by combining the cytoplasmic male sterility (CMS) line ‘Guoqing No.1’ (G1) Shatian pummelo with Hirado Buntan pummelo (HBP) through protoplast fusion. As a mitochondrial cybrid, G1 + HBP inherits mitochondria from the CMS donor G1 and the nuclear and chloroplast genomes from HBP, resulting in male sterility (Jiang et al. 2023). Mitochondrial cybrids, G1 + HBP, therefore provide an efficient pathway to incorporate complex traits, including seedless, drought and disease resistance into commercial citrus varieties in a shorter time frame.

In wheat, the cultivar Shanrong 3 demonstrates a distinct strategy that relies on the introgression of chromosomal fragments through somatic hybridization. Small chromosome segments from *Thinopyrum elongatum*, a wild relative known for its drought, salinity, and disease tolerance, were incorporated into common wheat, conferring high yield potential and strong salt tolerance. Molecular analyses further revealed that Shanrong 3 carries the *TaWD40‐4B.1C* allele, which enhances catalase activity and mitigates oxidative damage under drought conditions (Tian et al. 2023). This functional allele directly contributes to stress resilience, providing a genetic basis for both yield stability and environmental adaptability.

In Banlangen rapeseed (*B. napus* × *Isatis indigotica*, Songyou No.1), intertribal somatic hybrids between *B. napus* (AACC) and *I. indigotica* (II) were produced via protoplast fusion. Subsequent backcrossing generated monosomic alien addition lines, each carrying a single chromosome of *I. indigotica* within the *B. napus* background (Kang et al. 2014). This line successfully introduced genes for disease resistance, stress tolerance, and cytoplasmic male sterility from *I. indigotica* while preserving the agronomic performance of *B. napus*.

These approaches, cytoplasmic fusion, chromosomal fragment introgression, and whole‐chromosome addition, provide complementary and powerful tools for engineering crops with enhanced resilience to the escalating challenges of global climate change. When integrated with modern genomic selection and genome editing technologies, these methods hold great promise for accelerating the deployment of climate‐resilient cultivars across diverse agricultural systems.

#### 4.1.2 Protoplast Assays as a Versatile Platform for Dissecting Plant Stress Responses

Protoplast assays provide a fast and precise way to interrogate plant stress biology at the single‐cell level. Because cell walls are removed, stimuli and probes reach the plasma membrane and intracellular compartments efficiently (Yoo et al. 2007), and readouts such as luciferase reporters (Yoo et al.^2007^), ratiometric sensors (Andres et al. 2024), calcium dyes (Qiu et al. 2020), and immunoblot markers (Wang et al. 2021) can be quantified within hours. Over the past decade, this system has been used to reconstruct stress signaling modules (Asai et al. 2002; Cheng et al. 2015), measure kinase cascades (Asai et al. 2002; Cheng et al. 2015), visualize ion and reactive oxygen species dynamics (Dai et al. 2022), and even select stress‐tolerant regenerants (Kiełkowska et al. 2019), giving mechanistic and translational insights that complement whole plant studies.

Abiotic stress signaling, especially abscisic acid responses to drought and osmotic stress, illustrates the strengths of protoplast‐based reconstruction (Fujii et al. 2009). In rice green tissue and *Arabidopsis* mesophyll protoplasts, the core ABA module consisting of PYL or RCAR receptors, clade A PP2Cs, SnRK2 kinases, and ABRE binding bZIP transcription factors can be reconstituted to drive ABA dependent promoters with tunable component stoichiometry. These assays let researchers test receptor coreceptor combinations, quantify kinase outputs, and map negative regulation by PP2Cs, thereby clarifying how ABA sensitivity emerges from network composition. Results from these transient systems align with drought relevant phenotypes in intact plants, underscoring physiological relevance (Kim et al. 2015).

Endoplasmic reticulum (ER) stress and the unfolded protein response (UPR) can also be captured cleanly in protoplasts (Carrillo and Christopher 2022). A rapid protocol uses tunicamycin or dithiothreitol to trigger ER stress, then detects UPR activation via bZIP60 splicing or UPR reporter upregulation in protoplasts (Liang et al. 2023). Because the assay collapses tissue heterogeneity, it is well suited for dissecting pathway hierarchy, quantifying dose‐response relationships, and screening effectors that modulate proteostasis under heat or osmotic stress contexts.

For biotic stress, protoplasts enable high‐resolution analysis of pattern recognition receptor signaling. Upon treatment with the bacterial flagellin peptide flg22, *Arabidopsis* protoplasts show rapid MAPK activation and substrate dynamics (Sheikh et al. 2016), including disruption of the MPK6–ERF104 complex, a response that can be tracked by fluorescent protein reporters (Bethke et al. 2009; Asai et al. 2002). Chitin perception has been clarified by demonstrating that the LysM receptor kinase LYK5 binds chitin with much higher affinity than CERK1 and forms a ligand‐induced complex with CERK1, events that can be tested by co expression and interaction assays in the protoplast system (Cao et al. 2014). Downstream, a PAMP‐triggered MAPK cascade phosphorylates lipid kinase PIP5K6 in mesophyll protoplasts, reshaping phosphoinositide pools and coupling receptor signaling to membrane trafficking and reactive oxygen species production (Menzel et al. 2019). Together these cell‐level data map the early seconds to minutes of innate immune signaling that are difficult to resolve in whole tissues.

Beyond pathway mapping, protoplasts support quantitative imaging of ion and redox dynamics during stress. Calcium influx can be recorded with fluorescent indicators (Qiu et al. 2020) or genetically encoded sensors after elicitor or osmotic challenge, while ROS bursts can be measured by dyes or reporter constructs (Cheng et al. 2015; Maintz et al. 2014). These readouts integrate with transient expression to test how specific receptors, kinases, or transporters shape the amplitude and timing of ionic and oxidative signals that drive acclimation (Boudsocq et al. 2010).

Finally, protoplast culture can link cell level stress exposure to regenerative outcomes (Jin et al. 2020; Tiew et al. 2015). In carrot, NaCl imposed directly on protoplast cultures yielded regenerants that survived saline soil better than controls, demonstrating that single cell selection can produce stress‐tolerant plants and offering a route to complement genomic prediction or gene editing in crop improvement (Kiełkowska et al. 2019).

#### 4.1.3 Protoplast Regeneration and Gene Editing for Stress‐Tolerant Crop Breeding

Through protoplast assays, researchers have uncovered many genes involved in plant stress responses. Yet, because traditional breeding methods sometimes fail to deliver the anticipated traits, plant biologists increasingly focus on dissecting the causal genes that directly govern these phenotypes. Genetic perturbation of these genes, through overexpression or targeted mutations, can generate desired traits in a more precise and controlled manner. This genetic perturbation approach eliminates the need for hybridization or cell fusion, which are generally limited to closely related species. *Agrobacterium*‐mediated genetic transformation was first applied to the protoplast platform, the transformation of single cells, but the efficiency was too low to be practical (Márton et al. 1979; Wullems et al. 1981a, 1981b). The successful implementation of *Agrobacterium*‐mediated genetic transformation on multiple cell platforms (Hwang et al. 2017), such as callus, in numerous crops provided an efficient method for such genetic perturbation. However, such multi‐cell transformation methods often regenerate transformed plants from multicellular tissues (Kononov et al. 1997; Gao 2021), increasing the risk of chimeric individuals containing a mix of edited and unedited cells (Scintilla et al. 2022; Figure 3). The successful development of high‐efficiency PEG/Ca^2+^‐mediated chemical transfection on protoplasts (Sheen 2001; Yoo et al. 2007; Wu et al. 2009; Lee et al. 2012) to deliver the gene engineering tools, enables the introduction of genes or mutations in single cells. Protoplast‐based regeneration originates from a single cell, allowing for the generation of fully edited, non‐chimeric plants with uniform genetic backgrounds (Figure 3; Woo et al. 2015; Andersson et al. 2017; Lin et al. 2018; Hsu et al. 2019; Hsu, Lee, et al. 2021; Hsu, Yuan 2021; Lin, Hsu, et al. 2022; Wu et al. 2022; Scintilla et al. 2022; Li et al. 2023; Su et al. 2023; Wu et al. 2023; Gambino et al. 2024; Hsu et al. 2024).

As one of the most effective tools in modern genetic engineering, the CRISPR/Cas system enables precise, targeted genome modifications, thereby accelerating efforts to link gene function with plant adaptation and environmental responses. Comparison with previous gene editing tools, this system is particularly advantageous due to its precise target‐site recognition, ease of delivery into host cells, and capability for targeted insertions, deletions, and substitutions (Gao 2021). CRISPR‐based genome editing has now been widely applied beyond the model plant *A. thaliana* to a broad range of economically and biologically important crops. It is used for diverse purposes including gene knockouts, precise DNA insertions (knock‐in), microRNA functional studies, peptide‐related gene editing, and trait improvement. Introduction of CRISPR/Cas gene‐editing constructs via *Agrobacterium*‐mediated transformation or particle bombardment has been widely used in research on enhancing crop resistance to both biotic and abiotic stresses. For example, mutating *TaWRKY19* regulates the production of reactive oxygen species, thereby enhancing resistance to wheat stripe rust (*Puccinia striiformis* f. sp. *tritici*, Wang et al. 2022); knockout lines of *EIN2L*, *EIL3*, and *EIL4* in soybean improve water use efficiency, thereby increasing drought sensitivity (Ban et al. 2025); loss‐of‐function mutations in the *SQUAMOSA promoter‐binding‐like factor* (*OsSPL8*) gene in rice enhance tolerance to herbicide, drought, and salt stress (He et al. 2025); and *Agrobacterium*‐mediated delivery of CRISPR/Cas constructs targeting *MsWOX13‐2* in alfalfa improves flooding tolerance (Subedi et al. 2025). Editing cryptochrome 1 in poplar promoted wood formation (Chen, Fan, et al. 2024), while YFP knock‐in at *P5CS1* and *AFL1* enabled tagging of stress‐related proteins (Longkumer et al. 2024). CRISPR has helped uncover key genes involved in disease susceptibility (Liu, Zhang, et al. 2024; Qi et al. 2024), drought and salt tolerance (Liu, Wu, et al. 2024; Zhang et al. 2024; Li, He, et al. 2024), hormone signaling (Ren et al. 2024), nodulation (Li, Wang, et al. 2024), and cold response (An et al. 2024). It has also advanced the study of microRNAs and microRNA‐encoded peptides in responses to metal stress and viral infection (Lu et al. 2024).

Collectively, these studies highlight CRISPR as a powerful and versatile platform for plant functional genomics and crop improvement, especially for stress tolerance, underscoring its rapid expansion and versatile utility in plant biology and crop improvement. These modifications can effectively mimic natural evolution, spontaneous mutations, or hybridization events and can be achieved within one to two generations (Gao 2021; Yu, Tu, et al. 2021; Su et al. 2023; Hsu et al. 2024). With the rise of CRISPR, genetic perturbation has progressed from a limited tool to a powerful and versatile method for introducing genes and mutations (Gao 2021; Li, Sun, et al. 2024). In terms of technological development in this area, two conceptual approaches can be distinguished: one that allows for selfing‐based breeding (Inbred propagated crops) and another that does not (Asexual propagated crops; Figure 3). Selfing‐based breeding relies on two essential conditions: (1) a sufficiently short life cycle, and (2) the ability to undergo multiple generations of selfing. Protoplast‐based technologies are highly beneficial to both approaches, as outlined below.

Let us first consider the second approach—Asexual propagated crops (like grapes), cases in which selfing‐based breeding is not feasible (Figure 3A). This includes species that have long life cycles, such as woody perennials (Wang et al. 2023), or are genetically constrained, such as polyploid crops like *Musa* spp. and *S. tuberosum* that are incapable of producing seeds for propagation (Andersson et al. 2017; Cheng et al. 2024), or species in which selfing leads to the accumulation of deleterious genetic mutations (Goldberg et al. 2010). In these situations, traditional selfing is either impractical or biologically impossible, making alternative strategies necessary for trait fixation and improvement.

When it comes to the incapability of selfing, the issue is binary—either selfing is possible or it is not. If selfing is not possible, there is no way to achieve a new variety through traditional breeding that carries a single trait modification while retaining the rest of the elite parental genetic background. In such cases, new technologies are required to turn a definitive ‘no’ into a ‘yes.’ In contrast, a long‐life cycle is not a binary issue, but rather a matter of degree. Selfing is theoretically possible, but the process is so time‐consuming that it becomes impractical. In this case, new technologies are needed to convert ‘long’ into ‘short,’ enabling more efficient breeding timelines.

Regarding the infeasibility of the selfing strategy, the major limitations are either the inability to produce seeds or the accumulation of deleterious mutations over successive generations of selfing (the yes or no issues). A platform based on asexual reproduction could solve both of these issues in one fell swoop. Protoplast regeneration provides an effective asexual reproduction platform that directly addresses the limitations of the selfing strategy. Through the isolation, genome editing, and regeneration of single cells, this approach allows for the precise introduction of genetic modifications without the need for seed‐based propagation. Because regenerated plants are derived clonally from a single edited cell, this method eliminates the dependence on selfing to fix traits and avoids the accumulation of deleterious mutations across generations.

The challenge of long generation times, referred to here as the ‘long or short’ problem, is further amplified in crops with high genomic heterogeneity. Grapevine is a representative example. Introducing a green skin phenotype into an elite purple‐skinned cultivar presents considerable genetic and logistical difficulties. With a haploid chromosome number of *n* = 19, each parent can generate 2¹⁹ (524 288) unique gametes. Crossing two such varieties results in over 2³⁸ (> 274 billion) possible offspring (Figure 3A, upper left), making it extremely difficult to recover progeny that retain both the desired green skin allele and the elite genetic background. The situation is further complicated by grapevine's perennial growth habit and extended juvenile phase, which significantly slow down breeding cycles and increase resource demands. Genome editing offers a more targeted and potentially faster alternative. CRISPR‐based methods enable precise gene knock‐in or knock‐out; however, they often involve delivering CRISPR constructs into multicellular explants, resulting in chimeric tissues (Scintilla et al. 2022). Furthermore, to meet biosafety and regulatory requirements, the CRISPR transgenes must typically be segregated out to generate transgene‐free plants (Huang et al. 2016). In highly heterozygous, long‐lived species like grapevine, recovering edited individuals with both the desired allele and a largely unchanged elite genome through sexual segregation is inefficient and time‐consuming (Figure 3A, middle). Protoplast systems offer an attractive alternative. They allow transient transfection of multiple constructs into single cells, enabling expression of various proteins or RNAs simultaneously (Lee et al. 2012; Hsu et al. 2019). This flexibility facilitates multiplex gene editing without vector size constraints (Hsu et al. 2019), accelerating trait stacking without reliance on multi‐generational breeding. Although vector‐based delivery is technically straightforward, introducing large amounts of plasmid DNA increases the risk of random integration into the nuclear genome (Lin et al. 2018). Recent advances in CRISPR delivery now enable DNA‐free genome editing by introducing pre‐assembled RNP complexes into protoplasts. This approach avoids foreign DNA integration, reduces off‐target effects, and preserves genome integrity. Returning to the grapevine example, DNA‐free genome editing via protoplasts can generate the desired genotype in a single experimental cycle (Figure 3A, right; Yu, Bekkering, et al. 2021; Meyer et al. 2022; Lin,Hsu, et al. 2022; Hsu et al. 2024), offering a faster, more precise route to trait improvement—particularly valuable for complex, perennial crops.

For the first approach—cases in which selfing‐based breeding is feasible (Inbred propagated crops; Figure 3B). Due to their sufficiently short life cycles and genetic stability across successive generations of selfing, these species are well‐suited for selfing‐based breeding strategies. This also means that breeders can more readily reduce genomic heterogeneity in these species through inbreeding and selection. When such excellent species are already highly compatible with both traditional and molecular breeding approaches, what additional value can the protoplast platform offer? First, increased speed and precision of genome editing. Even in species amenable to selfing, achieving transgene‐free homozygous edits typically requires multiple generations of crossing and selection (Huang et al. 2016). As shown in Figure 3B, introducing the blue flower color gene into a red‐flowered variety typically requires hybridization followed by multiple rounds of backcrossing with the elite red‐flowered parent. Through repeated selection, the original red flower gene (*R*) can eventually be replaced by the blue flower gene (*B*), but this process often takes several years (Figure 3B, left). Alternatively, CRISPR reagents can be introduced into plants using multicellular explants for gene editing. However, this method is also time‐consuming due to the need to eliminate chimeric tissues and remove foreign DNA sequences (Figure 3B, middle). In contrast, protoplast regeneration enables the direct introduction of targeted modifications and immediate fixation of edited alleles at the single‐cell level: eliminating the need for generational turnover (Figure 3B, right; Huang et al. 2016). Second, higher efficiency in knock‐in strategies (Hsu, Yuan, et al. 2021). While *Agrobacterium*‐mediated transformation is technically feasible for gene knock‐in in many plant species, its efficiency remains low due to the limited copy number of donor DNA that *Agrobacterium* can deliver (Dong and Ronald 2021; Miki et al. 2018; Mudgett et al. 2024). By contrast, PEG/Ca^2+^‐mediated transfection of protoplasts in a DNA‐free CRISPR platform allows for the delivery of an overwhelming amount of RNP complexes, resulting in substantially higher editing efficiency (Hsu, Yuan, et al. 2021). Third, improved stability of genotype transmission to the next generation. *Agrobacterium*‐based transformation often leads to genetic chimerism, where the edited cells used for genotyping do not represent the germline (Zheng et al. 2020). This discrepancy can result in edited genotypes that are not faithfully inherited by progeny. In contrast, protoplast editing is initiated from a single edited cell, ensuring that the resulting plant is genetically uniform and that its genotype is stably transmitted through the germline (Lin et al. 2018). Fourth, reduced risk of unintended off‐target effects. In *Agrobacterium*‐mediated approaches, the CRISPR machinery is integrated into the plant genome and can remain active over time, increasing the chance of off‐target edits occurring unpredictably (Zhang et al. 2018; Graham et al. 2020; Zhang et al. 2021). In contrast, DNA‐free editing in protoplasts delivers the CRISPR components as transient RNP complexes, which degrade shortly (few days) after introducing the desired edit—minimizing the potential for unintended modifications. Importantly, all four of these advantages apply to crops in which selfing is either feasible or not feasible. As a gene‐editing platform, protoplast‐based regeneration techniques provide solutions to key challenges, including low gene‐editing efficiency, prolonged juvenile phases, and self‐incompatibility, ultimately enabling the development of edited regenerative plants.

Therefore, protoplast regeneration has emerged as a powerful platform for genome editing and has already been successfully applied across multiple crop species to enhance tolerance to both biotic and abiotic stresses. *S. peruvianum*, the most diverse wild tomato relative, is a key source of resistance genes for multiple diseases and pests in modern tomato breeding, we used CRISPR to knock out multiple genes, including the gene‐silencing pathway genes *SpSGS3* and *SpRDR6*, as well as disease‐resistance‐related genes *SpPR‐1*, *SpSystemin*, and *SpMOL1*, successfully regenerating both diploid and naturally tetraploid plants (Lin, Hsu, et al. 2022). Knockout plants for *spsgs3* and *sprdr6* lacked gene silencing activity and showed reduced resistance to tomato yellow leaf curl virus.

*S. tuberosum* L. (potato) cultivation is frequently challenged by environmental stresses, especially pathogen infections. Using CRISPR RNP complexes delivered into protoplasts to edit the target genes *StEDS1* and *StPAD4*, researchers regenerated mutant plants with enhanced salicylic acid accumulation, thereby improving resistance to pathogenic fungi (Moon et al. 2022).

Not only in knock out, in tobacco, Li et al. (2023) applied a protoplast‐CRISPR/Cas9 system to replace two resistance‐related regions of the *N* gene in *N. tabacum* with homologous fragments from *N. alata*, resulting in TMV‐U1 resistance in T₀ regenerated plants. However, this type of gene replacement shows low efficiency when using *Agrobacterium*‐mediated transformation or the particle bombardment method (Jin et al. 2023).

The two parameters are found to be most critical across species: target‐site selection and Cas9 protein quality. First, target‐site choice has a profound impact on editing outcomes. For example, in *Salvia miltiorrhiza* (Hsu et al. 2024), two sgRNAs designed for *MYB28* (target 1 and target 2) consistently resulted in zero detectable edits, whereas other target sites within the same gene achieved editing efficiencies of approximately 40%. This illustrates that even within a single locus, sgRNA performance varies dramatically and must be empirically validated. Second, we emphasize the importance of Cas9 purity, which is often overlooked in protoplast‐based editing. Commercial Cas9 preparations we tested did not support stable regeneration; protoplasts frequently lost viability or failed to form micro‐calli. By contrast, Cas9 purified in‐house using the protocol established by Prof. Steven Lin (Lin, Nguyen, et al. 2022) supported both high editing efficiency and successful regeneration.

As noted above, gene editing in woody perennials has been limited by long juvenile phases, making it difficult to obtain gene‐edited mutants. Recently, however, protoplast‐derived edited plants were regenerated from embryogenic calli of table grape cultivars (*Vitis vinifera* cv. Crimson Seedless and Sugraone), producing mutant lines of the powdery mildew susceptibility gene *VviMlo6* as promising candidates for disease resistance breeding (Scintilla et al. 2022). In citrus, a globally important woody perennial fruit crop, improvement through conventional breeding can take decades due to its complex reproductive biology, highly heterogeneous genome, and long juvenile phase (Talon and Gmitter 2008). Mahmoud et al. (2022) employed cationic lipid nanoparticles to deliver CRISPR/Cas editing constructs targeting the *Nonexpressor of Pathogenesis‐Related 3* (*CsNPR3*) gene. Regenerated protoplast‐derived plants exhibited elevated levels of the disease‐resistance protein CsPR1. Using Cas12a/crRNA RNPs to edit the citrus canker, major global citrus threat, susceptibility gene *CsLOB1* in citrus protoplasts, researchers obtained 39 regenerated plants after a 10‐month regeneration period, achieving a 97.4% biallelic/homozygous editing efficiency in T₀ plants (Su et al. 2023). These protoplast‐regenerated, RNP‐edited citrus plants with enhanced canker resistance have already been approved by USDA APHIS and are exempt from regulation by the U.S. Environmental Protection Agency (EPA).

Not only in woody plants, but protoplast regeneration in monocot crops is also extremely difficult (Table S3). In contrast to many dicot species, where explants grown in vitro can be directly used as the source for protoplast isolation, monocot species—whether Poaceae crops such as rice and wheat, or important fruit crops like banana—require the prior establishment of regenerable suspension cells and vigorous feeder cells. Only after embedding these cells can regeneration potentially be achieved (Abdullah et al. 1986), which greatly increases the technical complexity. Despite our successful experience in dicot species, we have not yet been able to establish a protoplast regeneration system in rice.

The successful regeneration of protoplasts into whole plants is influenced by multiple factors, including species‐specific cultivation environments, inorganic salt formulations, growth regulator types and application ratios, osmotic regulator compositions and concentrations, and culture medium composition for cell and tissue regeneration. The developmental trajectory of cells and tissues is highly dependent on expert evaluation of the specific requirements at different developmental stages (Wu et al. 2022; Wu et al. 2023).

Although regeneration has been reported in multiple species, its efficiency remains highly genotype dependent. These two observations are not contradictory. First, the overall hormonal logic of regeneration is relatively conserved across angiosperms. Most published work supports a general pattern in which auxin promotes callus initiation, followed by cytokinin‐driven shoot formation. However, this hormonal framework alone is not sufficient to guarantee successful regeneration. Each species and even each genotype exhibits its own sensitivity to auxin and cytokinin, differences in endogenous hormonal balance, and variation in cell totipotency. These factors collectively create large differences in regeneration success. Second, even well‐studied reference genotypes demonstrate the challenge. For example, recent assessments of *Arabidopsis thaliana* Col‐0 show that regeneration remains inefficient and difficult to reproduce despite decades of optimization (Jeong et al. 2021). This observation mirrors our own experimental experience. Overemphasizing specific molecular mechanisms such as plant growth regulators may inadvertently give the impression that regeneration is predictable once a single pathway is understood. Current evidence suggests that regeneration success emerges from a combination of hormonal responses, metabolic states, chromatin accessibility, and genotype‐specific cellular competence, and these factors vary widely across crops.

To address this complexity in practice, we follow a tiered strategy when establishing regeneration protocols. We begin with the simplest medium that works reliably in tobacco. If regeneration fails, we search the literature for established protocols. If no suitable method exists, we design empirical tests to identify optimal conditions. Our work in *S. peruvianum* illustrates this process. The first result in Lin, Hsu, et al. 2022 describes how we systematically evaluated medium components and hormone combinations before identifying a successful protocol.

### 4.2 Challenges in Crop Gene Editing: Technical and Regulatory Considerations

#### 4.2.1 Technical Challenges

Precise genomic insertion is facilitated through homology‐directed repair (HDR)‐mediated gene editing, which enables error‐free modification by utilizing a donor DNA template containing homologous arm sequences to transfer the donor sequence into the target locus. However, HDR occurs with low efficiency in higher plants. A study involving *N. tabacum* protoplasts, published in 1988, reported homologous recombination efficiencies as low as 1 in 10 000 (Paszkowski et al. 1988), and the persistently low efficiency of HDR continues to hinder the achievement of precise plant genome engineering (Barakate et al. 2020). The development of an efficient DNA sequence insertion platform using CRISPR/Cas gene editing technology remains a key objective for plant molecular breeders (Miki et al. 2018; Lu et al. 2020; Zong et al. 2022; Mudgett et al. 2024; Sun et al. 2025). Protoplast regeneration technology, which allows for the manipulation of high‐copy donor DNA, optimization of culture conditions, and high transfection efficiency, holds significant potential as a platform for the identification and generation of plants with precise gene targeting. The primary DNA repair pathway during the G1, S, and G2 phases of the cell cycle is non‐homologous end joining, while HDR repair predominantly occurs during the late S and G2 phases (Hiom 2010; Puchta and Fauser 2014). In 2021, Hsu and colleagues improved protoplast isolation by manipulating culture conditions with simple growth regulator formulations, thereby increasing the number of protoplasts in the late S and G2 phases of the cell division cycle. By co‐introducing easily synthesized, short, unmodified single‐stranded DNA donors along with CRISPR/RNP complexes into protoplasts, they enhanced the likelihood of gene targeting repair (Hsu, Yuan, et al. 2021). Notably, without the need for selectable marker genes, *N. benthamiana* target loci achieved up to 50% insertion efficiency, with one protoplast‐regenerated plant exhibiting precise sequence insertion. Additionally, in the model plant *B. oleracea*, a rapid‐cycling member of the Brassicaceae family, a 13.6% insertion efficiency was achieved. The targeted insertion sequences in these protoplast‐regenerated plants were inherited in subsequent generations (Hsu, Yuan, et al. 2021). However, only one case showed precise insertion of the six nucleotides at the intended site. Most of the others resulted in partial or repetitive, imprecise insertions.

By employing growth regulators, high‐copy donor DNA, CRISPR/RNP complexes, and other genome‐editing nucleases, in combination with simple transfection techniques, protoplasts present significant potential for achieving targeted DNA insertion across various plant materials. This approach holds promise for the precise insertion of functional genes in the future. However, to fully realize this potential, it will be essential to address key challenges, including improving cell cycle consistency, optimizing protoplast regeneration conditions, and preserving the integrity of long‐chain donor DNA within cells, preventing degradation by host cell mechanisms. Overcoming these obstacles will position protoplast‐based genome editing as a transformative tool for plant breeding.

#### 4.2.2 Regulatory Considerations

Currently, most crop gene‐editing strategies rely on *Agrobacterium*‐mediated transformation and biolistic (gene gun) that randomly introduce genetic modifications into the genome. Edited plants then undergo crossbreeding with wild‐type strains to eliminate foreign DNA sequences, producing gene‐edited varieties. While the United States and Japan have approved the commercialization of select gene‐edited crops, treating them as equivalent to conventionally bred varieties, *Agrobacterium*‐mediated transformation and gene gun methods frequently result in the unintended integration of vector backbone sequences into the host genome (Kononov et al. 1997; Gelvin 2003; Fu et al. 2000; Xu et al. 2024).

To avoid confusion between biological inputs and regulatory terminology, we distinguish three categories of genome editing workflows: (i) Transgene‐based editing. Editing components (Cas nuclease and sgRNA) are delivered as DNA constructs that can integrate into the genome. This approach introduces foreign DNA and is typically classified as GMO if the introduced sequences cannot be eliminated from the genome through selfing or backcrossing with the wild type. (ii) DNA‐free but not component‐free editing (CRISPR–RNP editing). Cas proteins and sgRNAs are delivered directly as ribonucleoprotein complexes. No foreign DNA integrates into the genome, but foreign proteins and RNA molecules are transiently present in the cell. Many regulatory jurisdictions treat these products differently from transgenic organisms, yet they are not strictly ‘foreign component‐free.’ (iii) Foreign component‐free editing. Editing is achieved through endogenous repair machinery with no exogenous nucleic acids or proteins remaining at any stage.

Regulatory policies differ considerably across regions. While many countries have approved the cultivation of genetically modified crops, stringent regulations govern their field trials and commercialization, particularly in the European Union and several Asian nations, where GMOs containing foreign DNA are subject to heightened scrutiny due to food safety regulations and consumer concerns. Additionally, crops exhibiting self‐incompatibility, asexual reproduction, or prolonged juvenile phases pose substantial challenges for traditional transformation techniques, as these approaches do not readily enable the inheritance of edited genetic sequences through successive generations.

## 5 Concluding Remarks and Perspectives

In recent years, protoplast technology has evolved from a niche experimental system into a versatile and powerful platform at the forefront of modern plant biotechnology, particularly for elucidating stress‐resilience mechanisms and advancing crop breeding. As a cell wall–free, totipotent system, the protoplast enables a wide range of applications that span from rapid gene function analysis to precision genome editing and single‐cell transcriptomics. Its ability to receive foreign DNA, RNA, proteins, and ribonucleoprotein complexes through non‐integrative and DNA‐free methods has positioned it as a key tool for developing transgene‐free genome‐edited crops. Furthermore, protoplast‐based regeneration from single cells ensures clonal uniformity, enabling precise genotype‐to‐phenotype connections while avoiding issues of chimerism commonly associated with traditional transformation techniques.

In parallel, protoplasts have become indispensable in the advancement of plant single‐cell omics. Their inherent single‐cell nature and compatibility with high‐throughput sequencing platforms have enabled researchers to dissect cell‐type identities, developmental lineages, and transcriptional dynamics in diverse tissues and species. From fundamental insights into xylem differentiation to the mapping of stress‐responsive gene networks, protoplast‐mediated single‐cell RNA sequencing has opened new windows into plant developmental biology and gene regulation. Together, these advances highlight the central role of the protoplast as a unifying system that bridges molecular manipulation, functional genomics, and regenerative potential—making it an essential engine for accelerating both basic research and translational applications in plant science.

We next outline three key priorities for advancing the protoplast platform. First, The need for a stable transformation system. While current protoplast technologies have proven highly effective for transient expression and gene editing, the establishment of a robust and efficient system for stable transformation remains a major unmet challenge. Transient assays—such as those used for CRISPR‐mediated genome editing—can be performed successfully using protoplasts without the need for DNA integration. However, the long‐term expression of transgenes, such as those encoding therapeutic proteins or trait‐enhancing regulators, or genes conferring adaptation to climate change, requires stable genomic incorporation. Conventional *Agrobacterium*‐mediated transformation is often inefficient in protoplasts and is further complicated by issues such as chimerism and tissue specificity. At present, protoplasts are the only single‐cell platform for plant transformation, yet the efficiency of achieving stable transformation through this route remains low and technically demanding. Developing a simple, reproducible, and high‐efficiency protocol for stable transformation in protoplasts would significantly expand their utility for synthetic biology, functional genomics, and biotechnological applications.

Second, improving the precision and efficiency of small‐fragment knock‐in for endogenous gene editing. Unlike the first challenge, which requires the development of a stable transformation system, this challenge focuses on genome edits that can—and ideally should—be accomplished through transient expression. The editing tools become dispensable once the desired edits are made, so transient transfection suffices, eliminating the need for stable chromosomal integration. In many cases, the aim is not to integrate large transgenes such as vaccine proteins, but to insert or replace small DNA fragments at precise genomic loci. Examples include tagging endogenous genes with fluorescent reporters (e.g., GFP), or inserting short cis‐regulatory elements in promoter regions to tune gene expression. In other scenarios, such as replacing a disease‐associated cis element, targeted sequence replacement is needed instead of simple insertion. These modifications require a high level of positional accuracy—where the inserted or replaced sequence must be integrated exactly at the desired site without disrupting adjacent genomic features. Because these small‐fragment edits typically lack selection markers, they also demand high editing efficiency to ensure the recovery of successfully modified cells. While protoplast‐based transient expression is well‐suited for delivering CRISPR/Cas and donor templates, the technical challenge lies in achieving reliable and precise knock‐in or replacement events without introducing off‐target effects or surrounding sequence disruptions. Addressing this challenge will enable high‐resolution, functional manipulation of the genome in a non‐integrative, selection‐free manner—thereby opening new possibilities for cis‐regulatory analysis, synthetic promoter design, and precise trait tuning in plants.

Third, improving the efficiency and accessibility of protoplast regeneration. A key prerequisite for successful DNA‐free genome editing is the ability to perform protoplast isolation, transfection, and regeneration in a coordinated and efficient manner. While the first two steps—protoplast isolation and transfection—are now routinely achievable in most laboratories, as supported by our meta‐analysis of over 1000 studies and the widespread use of protoplasts in single‐cell RNA‐seq research, the final and most limiting step—regeneration—remains a major bottleneck. Despite over 600 published references describing protoplast regeneration protocols (Yue et al. 2021), only a limited number of laboratories worldwide have successfully implemented full DNA‐free gene editing pipelines. Our data further confirm that, while protoplast isolation and transfection protocols can be readily re‐established across different species and tissue types, regeneration remains highly genotype‐dependent and condition‐sensitive, often requiring complex optimization that is difficult to reproduce across systems. Recent discoveries in developmental biology offer promising directions for overcoming this challenge. A growing number of genes related to cell proliferation, totipotency, and regeneration have been identified and functionally validated in the context of *Agrobacterium*‐mediated transformation (Bennur et al. 2025). These findings could be directly applied to enhance regeneration efficiency in protoplast systems. Not only does this offer significant value for applied biotechnology—by improving the feasibility of transgene‐free genome editing in diverse crops—but it also holds fundamental scientific importance. Protoplast regeneration provides a unique window into plant cellular reprogramming, enabling deeper investigation into totipotency, cell fate transitions, and developmental plasticity at the single‐cell level.

Protoplast‐based systems provide a powerful interface between current genome editing tools and future plant synthetic biology. Their single‐cell accessibility enables direct manipulation and evaluation of genetic circuits, regulatory elements, and metabolic pathways prior to stable transformation. Knowledge gained from protoplast transfection and regeneration is also driving the development of in planta delivery methods, including nanoparticle‐mediated cargo transfer and peptide‐assisted genome editing. Together, these advances position protoplast platforms as key enablers for precise, efficient, and genotype‐independent engineering of complex traits in plants.

## Conflicts of Interest

The authors declare no conflicts of interest.

## Supporting information


**Supplementary Figure 1:** Types of treatments and tools.
**Supplementary Figure 2:** Experimental purposes.
**Supplementary Figure 3:** Species.
**Supplementary Figure 4:** Tissue sources.

**Supplementary Table 1:** 1050 studies.

**Supplementary Table 2:** The research articles which identified xylem cells in their single‐cell transcriptomic datasets.

**Supplementary Table 3:** Regeneration conditions.

## Data Availability

The data that support the findings of this study are available on request from the corresponding author. The data are not publicly available due to privacy or ethical restrictions.
