# Supplementary material for: Protoplast‐Based Functional Genomics and Genome Editing: Progress, Challenges and Applications
Source: Plant Cell Environ. 2026 Jan 11;49(4):2183–99. doi: 10.1111/pce.70375 (PMC12976588; doi:10.1111/pce.70375)
Supplement: Supplementary file 1 — Supplementary Figure 1: Types of treatments and tools. Supplementary Figure 2: Experimental purposes. Supplementary Figure 3: Species. Supplementary Figure 4: Tissue sources. [file PCE-49-2183-s001.pptx]

## Slide 1
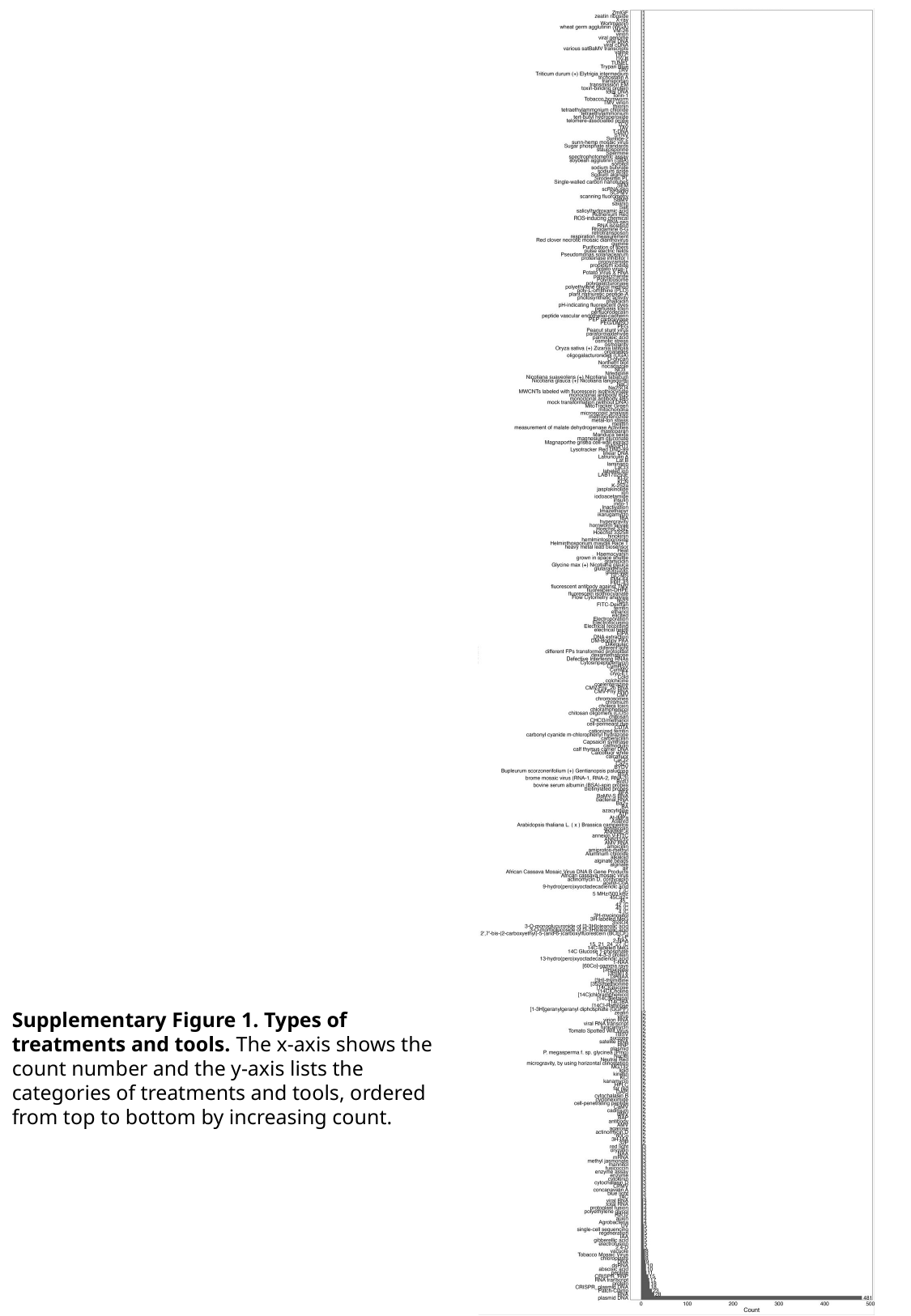

Supplementary Figure 1. Types of treatments and tools. The x-axis shows the count number and the y-axis lists the categories of treatments and tools, ordered from top to bottom by increasing count.

## Slide 2
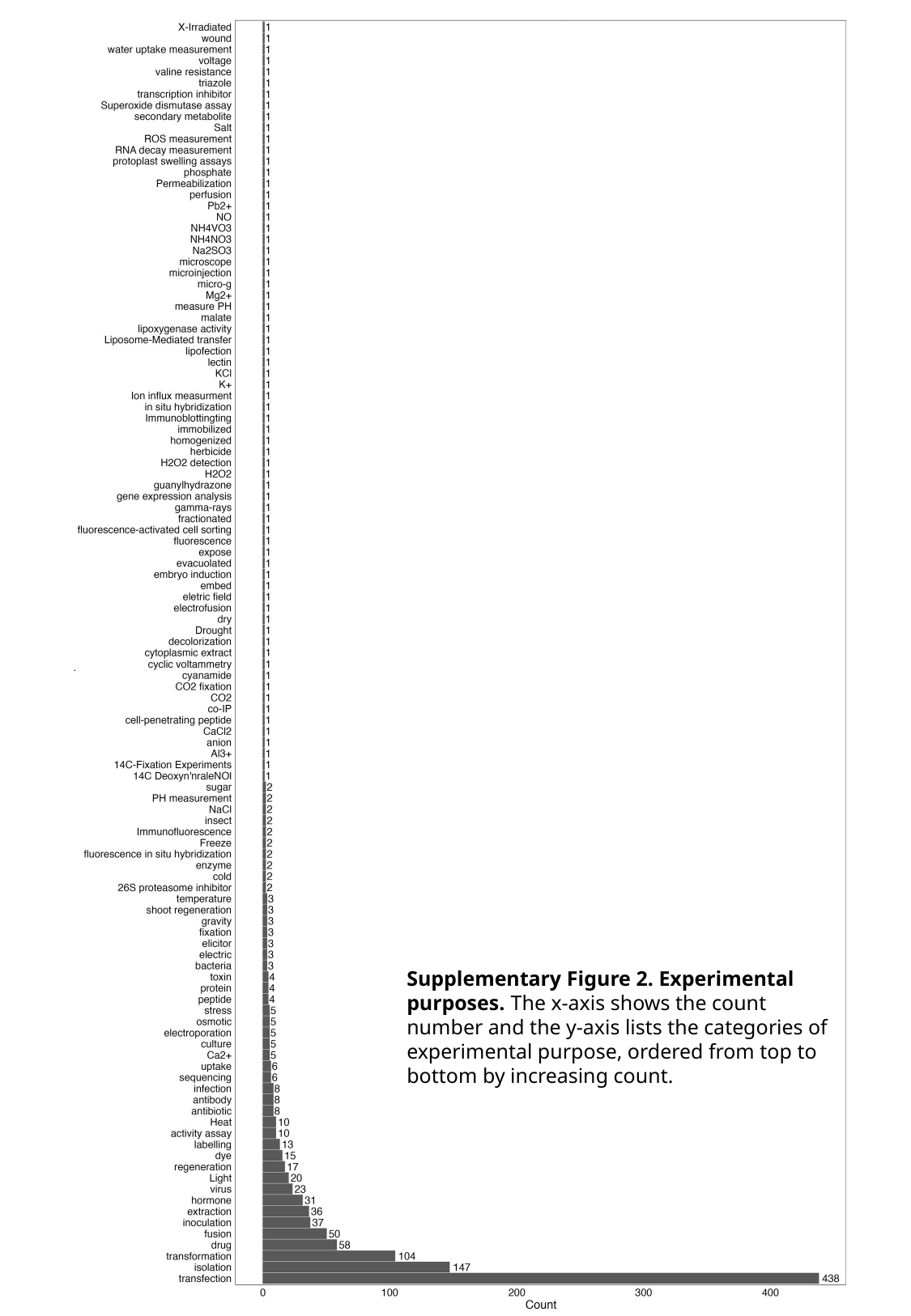

Supplementary Figure 2. Experimental purposes. The x-axis shows the count number and the y-axis lists the categories of experimental purpose, ordered from top to bottom by increasing count.

## Slide 3
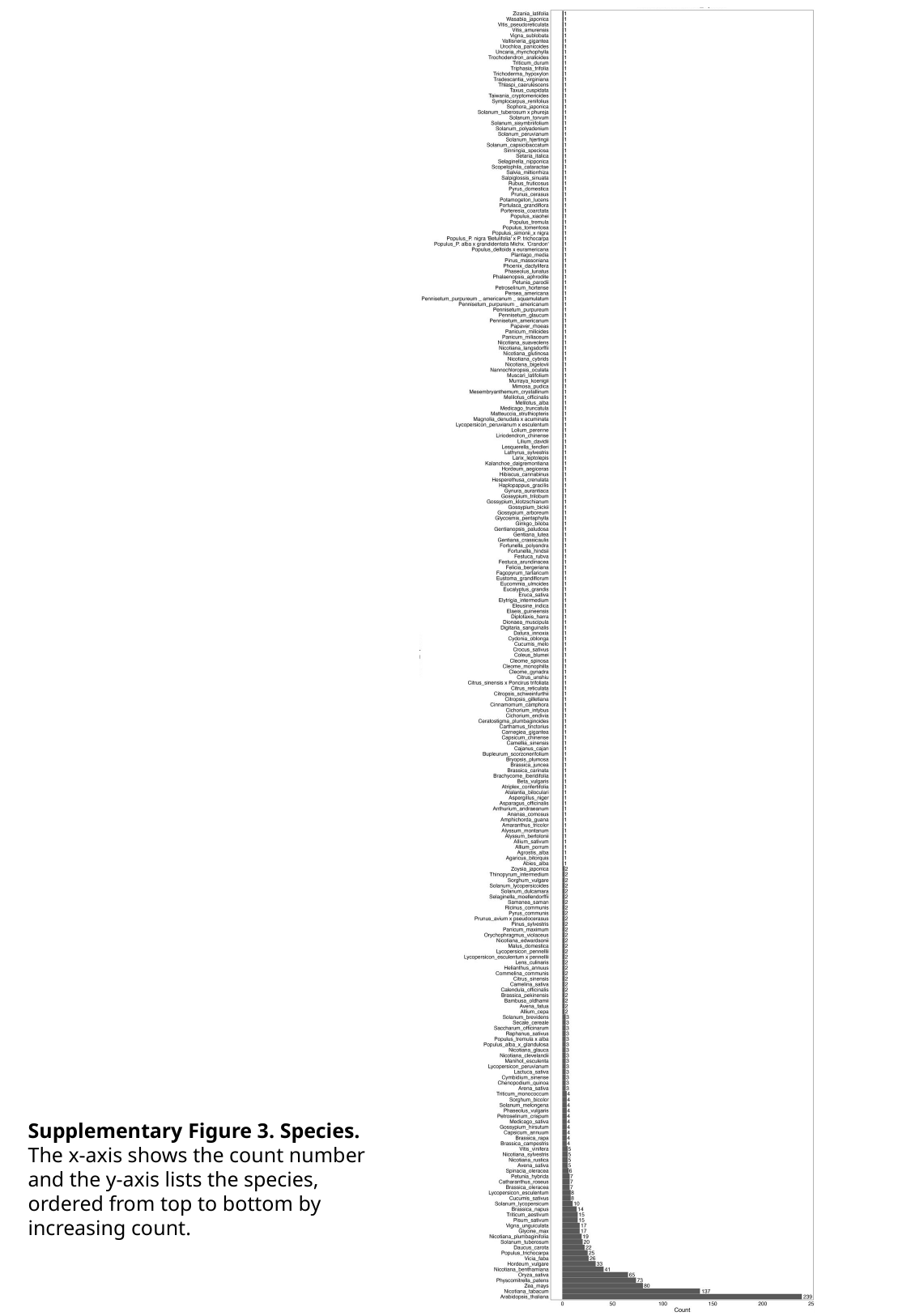

Supplementary Figure 3. Species. The x-axis shows the count number and the y-axis lists the species, ordered from top to bottom by increasing count.

## Slide 4
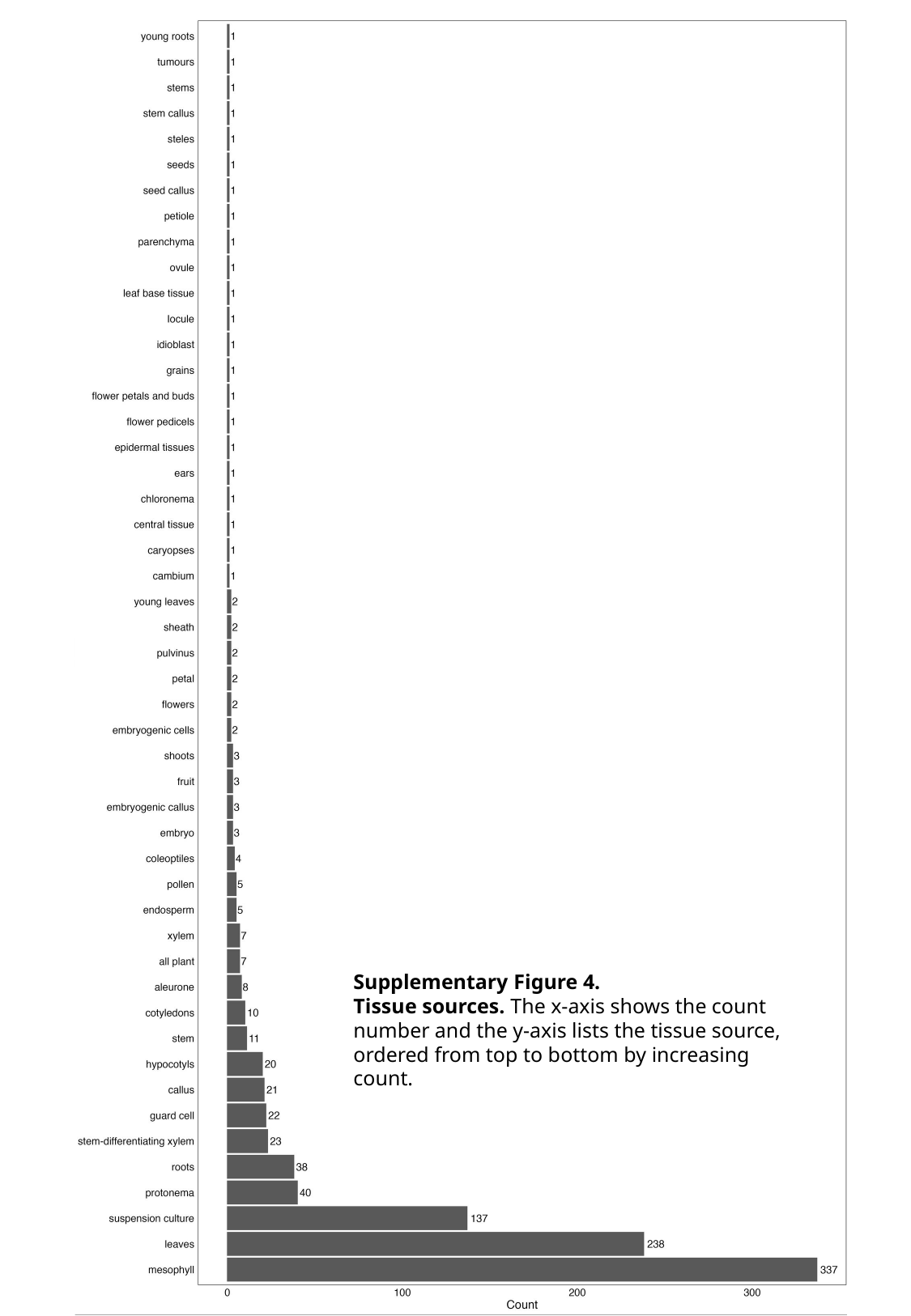

Supplementary Figure 4.
Tissue sources. The x-axis shows the count number and the y-axis lists the tissue source, ordered from top to bottom by increasing count.

## Slide 5
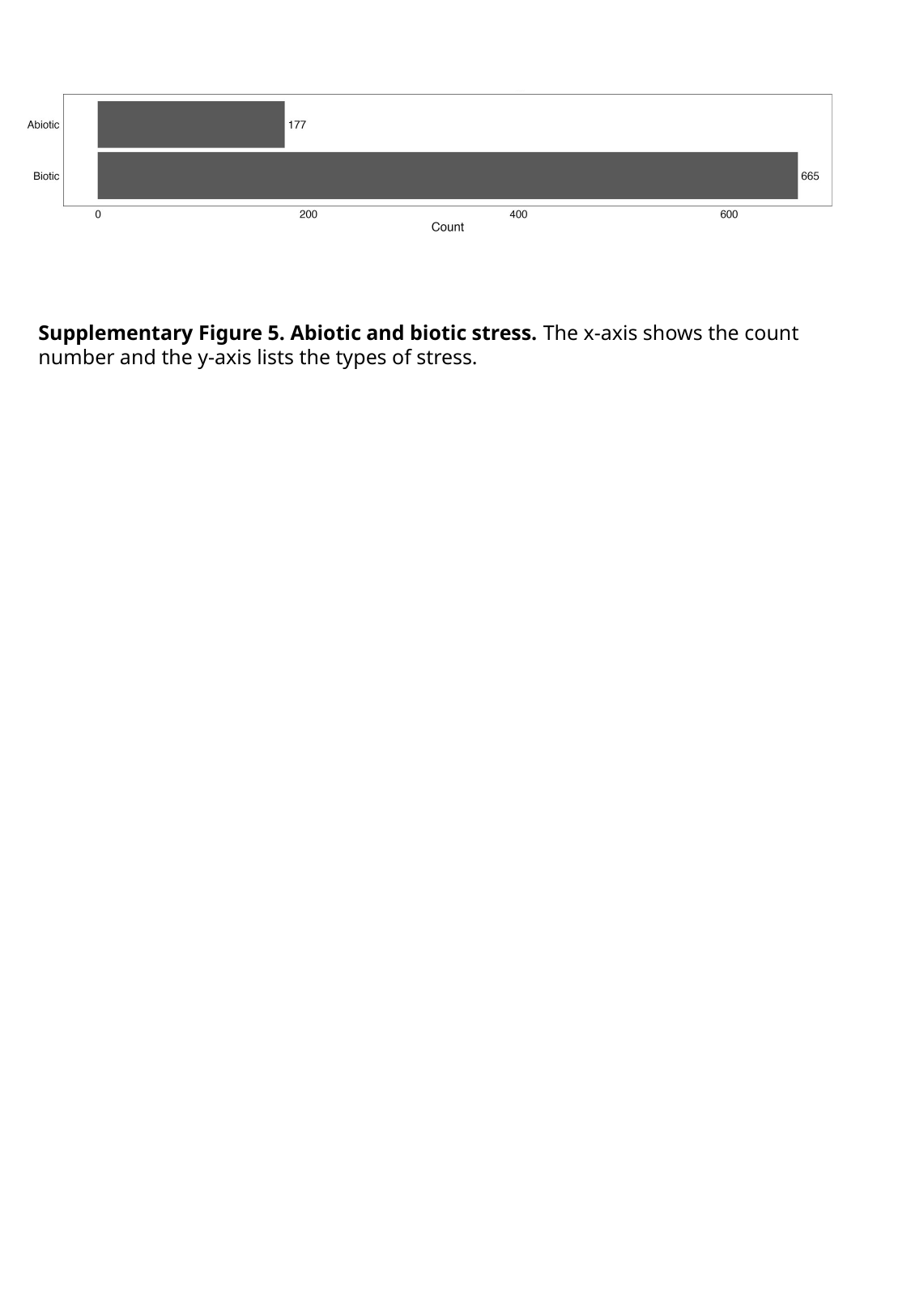

Supplementary Figure 5. Abiotic and biotic stress. The x-axis shows the count number and the y-axis lists the types of stress.
